# Pachy-Meningitis in a Monkey

**Published:** 1880-10

**Authors:** 


PACHY-MENINGITIS IN A MONKEY, OR WHITE-THIED COLOBUS—COLOBUS-BICOLOR—[Central Park Zoölogical Garden].

The following history was obtained from the Superintendent, Mr. William C. Conklin. The first symptom noticed was, that a few days before death the animal appeared dumpish and less playful than usual. A dose of Oleum Recini was administered, which operated well and temporarily relieved the stupidity of the monkey, but soon he relapsed into the former condition. Treatment was continued, but he gradually became worse and died. The day following his death a necropsy was made, by Dr E. C. Wendt in the presence of Superintendent Conklin and Dr. Porter. Rigor mortis not well marked. *Thoracic Cavity.*—A small amount of fibrine was found in the pericardial sac, otherwise the heart and lungs were normal. *Abdominal Cavity.*—Throughout the alimentary tract the mucus membrane was very loosely attached to the underlying coats and separated on the slightest provocation The other organs of the abdomen were normal. *Brain.*—When an attempt was made to remove the calvarium it was found firmly adherent to the dura-mater. These adhesions were so firm that the bones were broken in many places before they could be completely removed. The whole convexity of the brain was deeply congested. The brain substance proper appeared normal. From the history and the findings at the necropsy the case may be classed as one of Pachy-Meningitis.—Notes by W. H. P.

